# Implementing policies and predictive stochastic models to attend to borderline personality disorder crises: the dysthymia-suicide cycle

**DOI:** 10.1192/j.eurpsy.2024.1634

**Published:** 2024-08-27

**Authors:** C. G. Lazzari

**Affiliations:** Community Mental Health, UK NHS, BRIGHTON, United Kingdom

## Abstract

**Introduction:**

UK healthcare is undergoing significant challenges in facing borderline personality disorder (BPD) and accommodating the increased demand to allocate sufficient care and carers to deal with BPD’s growing number and emotional and suicidal crises.

**Objectives:**

To generate forecasting models and preventive policies to deal with BPD crises and improve the effectiveness of the UK National Healthcare Service in suicide prevention (NHS).

**Methods:**

The underlying analysis framework is stochastic forecasting. We used current knowledge and data to complete systematic future predictions extracted from recent trends. A logical-mathematical model generated the required expressions. The software for logic prediction and annotation was Wolfram Alpha (Wolframalpha.com).

**Results:**

Persons with BPD become suicidal because the team cannot comprehend and address the cycle of dysthymia, rumination and suicide. The BPD crises start from Stage 1 (α), assessing the comorbidity between BPD with dysthymia, cyclothymia, autism and ADHD. Teams shall avoid overmedication as ineffective. Stage 2 (β) is introspection and rumination, which do not respond to pharmacotherapy. The health carers establish if rumination is present and suggest distraction techniques. Stage 3 (γ) is when constant rumination with catastrophising leads to hopelessness. Stage 4 (δ) is when BPD starts feeling more anxious, depressed and unable to stop rumination. We suggest thought-stopping techniques and discourage social isolation, which triggers rumination. As BPDs use external locus of control and aim for higher dosages of antidepressants and anxiolytics with minimal effect, we explain that medication is not the only solution. Stage 5 (ε) is a crisis and panic attack because constant rumination brings back traumatic thoughts focused on the past, present and future. This is when BPDs self-refer to the hospital, attempt suicide, and feel that hospital admission is the only solution. The stages combined generated Model I. The Model II forecast Δ from this study is that we will observe a higher frequency (Δ) of hospital occupancy (Δbo = A), suicidal attempts (Δsa = B), and heavy service use (Δsu = C) by BPDs.

**Conclusions:**

The predictive model algorithm has thus extracted (1) *Model I* (Analysis): [α → (β → (γ → (δ → ε)))] = Z; The truth density for Model I and its strength of prediction for stage progression is 96.87% in the dysthymia-rumination-suicide cycle; and (2) *Model II* (Prediction): Z implies (A And B And C), Z→A ∩ B ∩ C; the truth density for the Model II is 56.25% for predicting a national shortage of healthcare resources. The combined models predict a truth of 73.81% in the outcomes of BPD crises in the UK NHS due to the dysthymia-suicide cycle.

**Disclosure of Interest:**

None Declared

